# Beneficial effect of methotrexate on a case of Nakajo-Nishimura syndrome

**DOI:** 10.1186/1546-0096-13-S1-P199

**Published:** 2015-09-28

**Authors:** K Kunimoto, F Ozaki, F Furukawa, N Kanazawa

**Affiliations:** 1 Wakayama Medical University, Dermatology, Wakayama City, Japan; 2 Kyoto University, Center for iPS Cell Research and Application, Kyoto, Japan

## 

Nakajo-Nishimura syndrome (NNS) is a very rare autosomal recessively-inherited autoinflammatory disorder that onsets in infancy with pernio-like rashes and gradually develops into partial lipodystrophy, accompanied with remittent fever and nodular skin eruptions. This disease is caused by a unique mutation of the *PSMB8* gene, which not only impairs an enzymatic activity of the encoding beta5i subunit, but also disturbs formation of the immunoproteasome complex. As the pathogenesis for NNS, cellular accumulation of ubiquitinated and oxidized proteins due to immunoproteasome deficiency is considered to cause MAP kinase activation with nuclear accumulation of phosphorylated p38 and following IL-6 production.

The treatment for Nakajo-Nishimura syndrome has not been established. Inflammatory attacks can temporarily respond to the oral administration of high-dose corticosteroid, but they easily recur by tapering the dose of corticosteroid. Furthermore, the high-dose corticosteroid therapy has various side effects such as growth failure in infancy. In our child case of NNS (Kunimoto *et al*, Dermatology 2013), additional administration of methotrexate (MTX) significantly decreased a frequency of febrile attacks, in comparison to the treatment with oral corticosteroid alone. Notably, effectiveness of MTX was previously described on some infant cases of CANDLE syndrome, another *PSMB8*-mutated proteasome-associated autoinflammatory syndrome (PRAAS). MTX is known to execute anti-inflammatory effects through inhibition of folic acid-dependent enzymes, including dihydrofolate reductase (DHFR) and aminoimidazole carboxamide ribonucleotide transformylase (ATIC). ATIC inhibition causes accumulation of intracellular aminoimidazole carboxamide ribonucleotide and inhibits AMP deaminase, to increase the production of adenosine. Adenosine can inhibit superoxide production of neutrophils and their attachment to endothelial cells. As preliminary results, increased ROS production has been observed in primary neutrophils of NNS patients, suggesting one of the points that MTX affects.

